# A systematic review for sudden cardiac death in hypertrophic cardiomyopathy patients with myocardial fibrosis: a CMR LGE study

**DOI:** 10.1186/1532-429X-14-S1-O101

**Published:** 2012-02-01

**Authors:** Nackle J Silva, Antonio T Paladino, Mark Doyle, Sahadev T Reddy, Geetha Rayarao, June Yamrozik, Ronald B Williams, Diane A Vido, Robert W Biederman

**Affiliations:** 1 Allegheny General Hospital, Pittsburgh, USA

## Summary

To our knowledge this represent the first attempt at a formal meta-analysis to demonstrate that SCD is predicted by LGE, not just VT/VF.

## Background

Hypertrophic cardiomyopathy (HCM) is a genetic disease that affects the cardiac sarcomere, resulting in myocardial hypertrophy and disarray. Affected patients have a predisposition for malignant ventricular tachyarrhythmias and, consequently, sudden cardiac death (SCD). In single center studies, late gadolinium enhancement (LGE) defined fibrosis has been linked to the substrate for VT/VF. However, despite innumerable investigations, SCD has not been definitely attributable to LGE. Explanations for this are believed to be related to insufficient statistical power.

## Methods

We performed an electronic search of MEDLINE, PubMED and CMR abstracts for original data published or presented between Jan 01 - Mar 11. Key search terms: HCM, LV fibrosis, SCD and LGE. Studies were screened for eligibility based on inclusion criteria: referral for CMR exam with LGE for HCM; and follow-up for incidence of VT/VF and SCD. Relevant studies were summarized and the following data were abstracted: socio-demographic information; study design; incidence of reported VT/VF; SCD. Categorical variables were evaluated between pt groups via Chi-square test.

## Results

A total of 64 studies were initially identified. Of these, 4 (6.3%) were identified and included (n=1,063 pts, range 202 to 424). Three prospective and 1 retrospective studies were included. LGE was detected in 59.6% of pts. Mean follow-up was 43±14mo. Age 22-90 yrs (63% male). As expected, the presence of myocardial fibrosis was associated with VT/VF (χ2=6.5, p<0.05; OR 9.0 (95% CI 1.2 to 68.7). Moreover, myocardial fibrosis strongly predicted SCD (χ2=6.6, p<0.05; OR 3.3 (95% CI 1.2 to 9.7).

## Conclusions

Despite single center CMR studies, LGE has consistently predicted VT/VF while prediction of SCD has remained paradoxically unlinked. Although the lack of studies meeting our criteria limited our ability to perform a comprehensive meta-analysis, we have been able to demonstrate for the first time that LGE-defined fibrosis is a predictor of SCD in patients with HCM. This observation now demands a multi-center RCT for confirmation but supports the consideration of a primary indication of AICD implantation in HCM with LGE.

## Funding

Internal.

